# “Beer Potomania” – A Syndrome of Severe Hyponatremia with Unique Pathophysiology: Case Studies and Literature Review

**DOI:** 10.7759/cureus.2000

**Published:** 2017-12-29

**Authors:** Muhammad Uzair Lodhi, Tahira Sabeen Saleem, Aaron R Kuzel, Dawood Khan, Intekhab Askari Syed, Umar Rahim, Hafiz Imran Iqbal, Mustafa Rahim

**Affiliations:** 1 Medical Student, Department of Medicine, Raleigh General Hospital, Beckley, Wv; 2 Department of Medicine, Raleigh General Hospital, Beckley, Wv; 3 Department of Emergency Medicine, Lincoln Memorial University-Debusk College of Osteopathic Medicine; 4 Internal Medicine, Raleigh General Hospital, Beckley, Wv; 5 Pre-Medical Student, Department of Sciences, Queens University of Charlotte, Nc; 6 Nephrologist, Department of Medicine, Raleigh General Hospital, Beckley, Wv; 7 Assistant Clinical Professor of Internal Medicine, West Virginia University School of Medicine

**Keywords:** beer-potomania, severe hyponatremia, osmotic demyelination syndrome, alcoholic beer, potomania vs. siadh, serum sodium concentration, osmolar load, dilutional hyponatremia

## Abstract

Beer potomania, a unique syndrome of hyponatremia, was first reported in 1972. It is described as the excessive intake of alcohol, particularly beer, together with poor dietary solute intake that leads to fatigue, dizziness, and muscular weakness. The low solute content of beer, and suppressive effect of alcohol on proteolysis result in reduced solute delivery to the kidney. The presence of inadequate solute in the kidney eventually causes dilutional hyponatremia secondary to reduced clearance of excess fluid from the body. Early detection of hyponatremia due to beer potomania in the hospital is necessary to carefully manage the patient in order to avoid neurological consequences as this syndrome has unique pathophysiology. We are reporting two cases, presenting to the emergency department with severe hyponatremia. After a detailed initial evaluation of the patients and labs for hyponatremia, a diagnosis of beer potomania was established in both cases. Considering the unique pathophysiology of beer potomania syndrome, the patients were closely monitored and treated appropriately to prevent any neurological sequelae.

## Introduction

The unique syndrome of hyponatremia in heavy beer drinkers (consuming five or more drinks per day) was first reported by Gwinup, et al. in 1972 [1]. Hyponatremia is a common electrolyte abnormality in hospitalized patients with a history of chronic alcoholism. As the study conducted by Liamis, et al. showed 17.3% of hospitalized chronic alcoholics have severe hyponatremia [2]. However, it has been crucial to quickly diagnose beer potomania in a chronic alcoholic presenting with severe hyponatremia, due to other concomitant causes such as malnutrition, the use of diuretics or antipsychotics, congestive heart failure and cirrhosis.

## Case presentation

Case 1

History and physical examination

A 59-year-old male with past medical history significant for alcoholism, chronic obstructive pulmonary disease (COPD), grand-mal seizures, and mood disorder was brought to the emergency department (ED) following a seizure earlier in the day. The patient denied any fall, head trauma or loss of consciousness. He had a significant history of alcohol abuse over the last 25 years, drinking nine to 10 cans of beer per day. As reported by the patient's family, he had poor dietary habits and eats one meal in a day, three to four days per week. He has a 40-year history of smoking one pack a day. The patient denied any intravenous drug abuse, recent travel, or any history of hepatitis. Review of home medications revealed that he takes Trazodone, Risperidone, and Phenytoin. He mentioned that he is compliant with his medications. He denied any excessive water intake, diarrhea, vomiting, cold or heat intolerance, and lower extremities swelling. He also denied headache, cough, hemoptysis, chest pain, fever, night sweats or weight loss.

Upon initial physical examination, the patient appeared malnourished, lethargic and in moderate respiratory distress. His vitals were as follows: blood pressure of 127/83 mmHg, heart rate of 110 beats per minute, respiratory rate of 20 breaths per minute, oxygen saturation of 85% on room air and had no fever. His body mass index (BMI) was 16. His pupils were equal, round and reactive to light. He appeared euvolemic. Cardiac auscultation revealed regular rhythm without murmurs or gallops, and audible S1 and S2. He had wheezing, ronchi and generalized decreased breath sounds bilaterally on lung examination. He had tenderness in the epigastric region without rebound tenderness, with normal bowel movements and no organomegaly. He was oriented to person and place, but not to time. On neurological examination, he appeared confused and deep tendon reflexes were unremarkable. He had no tremors or asterixis. His Glasgow score was 14.

Hospital course and differential diagnosis

His laboratory workup performed at the time of presentation in the ED is listed below (Table 1). Patient's urine toxicology returned positive for cannabinoids and opioids. A chest x-ray was unremarkable. Echocardiogram of the heart showed mild ventricular hypertrophy with no diastolic dysfunction. Computed tomography (CT) of the head without contrast, showed cerebral atrophy with no acute changes.

**Table 1:** Biochemical and hematologic studies ordered at the time of initial presentation in the emergency department.

Test	Result	Reference
White blood cells (WBC)	7.3 x 10^3^ µL	3.4-10.8 x 10^3^ µL
Hemoglobin (Hb)	13.6 g/dL	12.6-17.7 g/dL
Hematocrit (Hct)	40.70%	37.5-51.0%
Platelet count	354 x 10^3^ µL	150-379 x 10^3^ µL
Serum sodium (Na)	118 mmol/L	134-144 mmol/L
Serum potassium (K)	4 mmol/L	3.5-5.2 mmol/L
Serum chloride (Cl)	90 mmol/L	96-106 mmol/L
Serum bicarbonate	27 mmol/L	18-29 mmol/L
Blood urea nitrogen (BUN)	4 mg/dL	6.0-24 mg/dL
Creatinine	0.56 mg/dL	0.6-1.2 mg/dL
Serum glucose	129 mg/dL	65-100 mg/dL
Serum calcium	9.2 mg/dL	8.7-10.2 mg/dL
Serum phosphate	3.2 mg/dL	2.5-4.5 mg/dL
Serum magnesium	2.1 mg/dL	1.7-2.2 mg/dL
Aspartate aminotransferase (AST)	76 IU/L	0.0-40 IU/L
Alanine aminotransferase (ALT)	35 IU/L	0.0-44 IU/L
Total protein	7.3 g/dL	6-8.3 g/dL
Albumin	2.9 g/dL	3.5-5.5 g/dL
Alkaline phosphatase (ALP)	99 IU/L	39-117 IU/L
Total bilirubin	1.4 mg/dL	0.0-1.2 mg/dL
Direct bilirubin	0.2 mg/dL	0.0-0.3 mg/dL
International normalized ratio (INR)	1	≤1.1
Serum uric acid	3.4 mg/dL	3.4-7.0 mg/dL
Thyroid-stimulating hormone (TSH)	1.12 µIU/mL	0.45-4.5 µIU/mL
Serum osmolarity	259 mOsm/kg	275-295 mOsm/kg
Urine specific gravity	1.043	1.003-1.030
Ketones in urine	Trace	Absent

Regular protocol for acute COPD exacerbation was followed, and patient oxygen saturation and partial pressure O2 returned to normal. Urinary sodium and osmolarity was not checked in the ED. The patient was administered intravenous 1 L of 0.9% sodium chloride in the ED, together with intravenous thiamine, folic acid, magnesium sulfate, multivitamins, and chlordiazepoxide. Nephrology was consulted for hyponatremia of sodium 118 mmol/L and the patient was admitted to the medical unit.

The patient’s poor nutritional status, being on Risperidone and significant history of alcoholism raised concern of dehydration, syndrome of inappropriate antidiuretic hormone (SIADH) and beer potomania, respectively.

The urine osmolality was 72 mOsm/kg H2O, and urine sodium was 19 mmol/L. Low urine osmolarity and low urine sodium levels excluded the SIADH and cerebral-wasting syndrome as the cause of this patient’s hyponatremia [3]. The patient also denied drinking excessive water, which ruled out psychogenic polydipsia. Patient’s unremarkable physical examination, together with clear chest x-ray and no significant abnormality on echocardiogram, made it easier to rule out congestive heart failure. Considering the patient’s history (of chronic alcohol abuse and recent seizure), clinical presentation (of lethargy and malnourishment), lab values (of low serum osmolarity, urinary osmolarity, urinary sodium level), and absence of any other plausible explanation, directed us to establish the diagnosis of beer potomania syndrome [4-5].

In the next 16 hours following the administration of 1 L of 0.9% sodium chloride in the ED, the patient had a brisk diuresis of about 3 L. The patient's sodium went up from 118 mmol/L to 129 mmol/L, an increase of 11 mmol/L in 16 hours. A one-liter bolus of 5% dextrose water (D5W) was given and then the patient was started on banana bag with base solution of D5W. With this fluid sodium level improved back to 127 mmol/L at 24 hours of admission.

D5W infusion was adjusted every few hours according to change in serum sodium level to prevent rapid auto correction of serum sodium level which could lead to osmotic demyelination syndrome (ODS). Over the period of the next few days, the patient’s serum sodium levels stayed constant between 131 and 133 mmol/L. The patient was feeling comparatively less lethargic and he did not develop any neurological sequelae.

**Figure 1:**
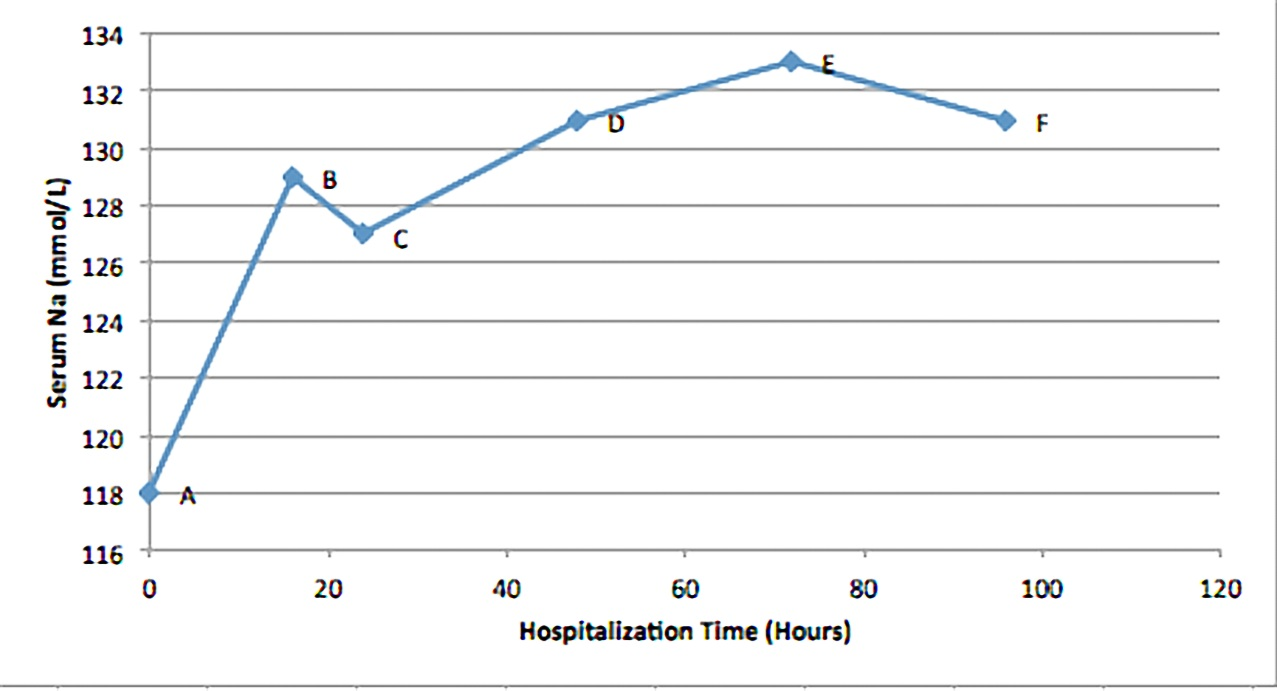
Serum sodium level (mmol/L) versus hospitalization time (hours). Point A, shows serum sodium level of 118 mmol/L on admission, when patient was started on 0.9% sodium chloride, together with thiamine, magnesium sulfate and folic acid. Point B, shows serum sodium level of 129 mmol/L at 16 hours after admission, when nephrology was consulted. 0.9% sodium chloride was discontinued, a bolus of D5W was administered followed by D5W based banana bag. Point C, shows serum sodium level of 127 mmol/L at 24 hours since admission. Point D, shows serum sodium level of 131 mmol/L at 48 hours. Point E, shows serum sodium level of 133 mmol/L at 72 hours, and point F shows serum sodium level of 131 mmol/L at 96 hours.

The serum sodium progression since admission in the ED until day five is shown below (Figure 1). The patient was also educated on alcohol cessation and suggested to slowly increase his dietary food intake.

Case 2

History and physical examination

A 60-year-old male presented to the emergency room with weakness and lack of appetite. It was accompanied with dizziness, but no loss of consciousness or trauma. The patient admitted drinking 12-20 cans of beer, 12 ounces each. He has been smoking two packs per day for the last 40 days. The patient denied taking any medicine at home. He denied any excessive water intake, diarrhea, vomiting, cold or heat intolerance, and swelling. He also denied headache, cough, hemoptysis, chest pain, fever, night sweats or weight loss.

Upon initial physical examination, the patient appeared malnourished, drowsy and unkempt. His vitals were as follow: afebrile, blood pressure of 96/67 mmHg, heart rate of 103 beats per minute, respiratory rate of 15 breaths per minute, oxygen saturation of 96% on room air. His BMI was 17.5. His pupils were equal, round and reactive to light. The patient appeared to have intravascular volume depletion with dry, pale skin and delayed capillary refill of about 4 seconds with low BP and tachycardia. Cardiac auscultation revealed regular rhythm without murmurs or gallops and audible S1 and S2. On lung examination, there were no rales, wheezes or crackles appreciated. His bowel sounds were normal with no tenderness, organomegaly or distention. He had no jugular venous distention or peripheral edema. He was oriented to person, place and time. On neurological examination, he appeared drowsy. Deep tendon reflexes were unremarkable. He had no tremors or asterixis. His Glasgow score was 15.

Hospital course and differential diagnosis

His biochemical and hematologic workup performed at the time of admission in the ED is listed below (Table 2). A chest x-ray was also done and it showed no signs of pulmonary edema or mediastinal mass. Echocardiogram of the heart was not performed. CT scan of the head without contrast showed no abnormalities.

**Table 2:** Biochemical and hematologic studies ordered at the time of initial presentation in the emergency department.

Test	Result	Reference
White blood cells (WBC)	9 x 10^3^ µL	3.4-10.8 x 10^3^ µL
Hemoglobin (Hb)	13.7 g/dL	12.6-17.7 g/dL
Hematocrit (Hct)	35.60%	37.5-51.0%
Platelet count	163 x 10^3^ µL	150-379 x 10^3^ µL
Serum sodium (Na)	106 mmol/L	134-144 mmol/L
Serum potassium (K)	4.6 mmol/L	3.5-5.2 mmol/L
Serum chloride (Cl)	74 mmol/L	96-106 mmol/L
Serum bicarbonate	24 mmol/L	18-29 mmol/L
Blood urea nitrogen (BUN)	9 mg/dL	6.0-24 mg/dL
Creatinine	0.4 mg/dL	0.6-1.2 mg/dL
Serum glucose	98 mg/dL	65-100 mg/dL
Serum calcium	7.9 mg/dL	8.7-10.2 mg/dL
Serum phosphate	3.7 mg/dL	2.5-4.5 mg/dL
Serum magnesium	1.9 mg/dL	1.7-2.2 mg/dL
Aspartate aminotransferase (AST)	43 IU/L	0.0-40 IU/L
Alanine aminotransferase (ALT)	69 IU/L	0.0-44 IU/L
Total protein	6.2 g/dL	6-8.3 g/dL
Albumin	2.7 g/dL	3.5-5.5 g/dL
Alkaline phosphatase (ALP)	123 IU/L	39-117 IU/L
Total bilirubin	0.9 mg/dL	0.0-1.2 mg/dL
Direct bilirubin	0.3 mg/dL	0.0-0.3 mg/dL
International normalized ratio (INR)	1	≤1.1
Serum uric acid	2.1 mg/dL	3.4-7.0 mg/dL
Thyroid-stimulating hormone (TSH)	1.13 µIU/mL	0.45-4.5 µIU/mL
Serum osmolarity	232 mOsm/kg	275-295 mOsm/kg
Urine random osmolality	159 mOsm/kg	300-900 mOsm/Kg of water
Urine specific gravity	1.012	1.00-1.030
Urine sodium	19 mmol/L	20-40 mmol/L

Considering the signs and symptoms of hypovolemia on physical examination, severe hyponatremia of 106 mmol/L and low-normal blood pressure, the patient was started on 0.9% sodium chloride-based banana bag in the emergency room. The patient also showed interest in food and had two big meals in the emergency room. A few hours after presentation, he had a brisk diuresis of unmeasured amount as he denied initially to have foley catheter. Within 16 hours of 0.9% sodium chloride-based banana bag fluid administration, the patient’s serum sodium jumped up to 119 mmol/L, an increase of 13 mmol/L. 0.9% sodium chloride-based intravenous fluid was discontinued but the patient’s serum sodium level went up to 128 mmol/L in first 32 hours since his presentation. Nephrology was consulted and the patient was moved to the medical unit.

**Figure 2:**
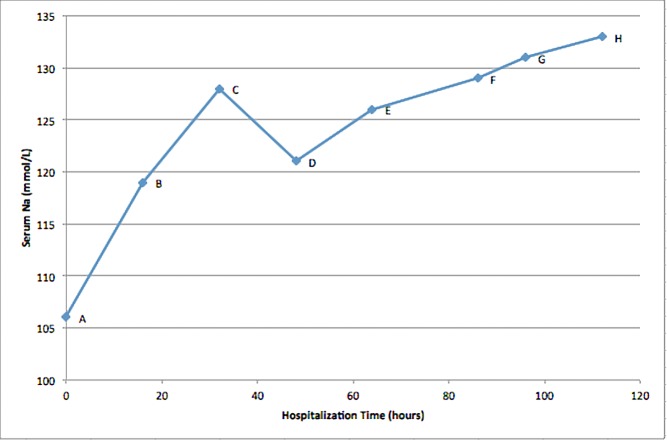
Serum sodium level (mmol/L) versus hospitalization time (hours). Point A, shows serum sodium level of 106 mmol/L on admission, when patient was started on 0.9% sodium chloride, together with thiamine, magnesium sulfate, folic acid and chlordiazepoxide. Point B, shows serum sodium level of 119 mmol/L at 16 hours after admission, 0.9% sodium chloride was discontinued. Point C, shows serum sodium level of 128 mmol/L at 36 hours since admission, nephrology was consulted at this point, 1 L bolus of D5W was given followed by D5W based banana bag. Point D, shows serum sodium level of 121 at 48 hours. Point E, shows serum sodium level of 126 mmol/L at 64 hours. Point F, shows serum sodium level of 129 mmol/L at 86 hours. Point G, shows serum sodium level of 131 mmol/L at 96 hours. Point H, shows serum sodium level of 133 mmol/L at 112 hours.

After admission to the medicine unit, the patient was started on adjusted amount of 5% dextrose water-based banana bag. Sodium level dropped to 121 mmol/L in the next 12 hours. The patient’s serum sodium level increased slowly over the next few days, as shown below (Figure 2). After day five onwards, the patient’s serum sodium stayed between 132 and 134 mmol/L and the patient did not develop any neurological sequelae.

The initial presentation of the patient pointed towards the hypovolemic hyponatremia, however, low serum uric acid could not be explained by simple hypovolemic hyponatremia. Given the patient’s low urine osmolarity and low serum uric acid, with poor oral intake and significant history of alcoholism raised the concern of beer potomania. Syndrome of Inappropriate ADH secretion (SIADH) and cerebral salt wasting syndrome were unlikely with low urine sodium and low urine osmolality. The initial rapid correction of hyponatremia with low serum uric acid level suggests that hyponatremia was contributed by both hypovolemic hyponatremia and beer potomania.

## Discussion

Pathophysiology

Understanding the pathophysiology is critical for the proper management of dilutional hyponatremia in a patient with beer potomania syndrome. A person with normal renal function and normal dietary intake removes about 600-900 mOsm/day. With the maximal urinary dilation of 50 mOsm/L, a person can excrete about 20 L of water without becoming hyponatremic, allowing for broad range of water intake (reaching up to 20 L) [5-7]. As free-water clearance in a person with normal diluting capacity is dependent on osmole excretion, a decrease in daily dietary osmole intake can have a vast decrease in the excretory capacity of the kidney. Therefore, this decrease in daily dietary osmoles in even minute fluid excess can cause dilutional hyponatremia. Beer potomania patients have a long-term history of beer intake, as well as a poor diet. Beer has trace amounts of sodium and almost negligible protein content. In addition, beer has some calories that prevent the muscular proteolysis resulting in a dramatic decrease in urea generation. Thus, these patients have very low osmolar load as dietary protein breakdown is the main component of the osmolar load, as well as small amounts from sodium and potassium. In our two cases, patients with nine to 20, 12 oz cans of beer daily, their approximate osmole intake was 225-250 mOsm/L per day. Assuming normal urinary dilation capability, any fluid intake more than 3-4 L will result in water retention and subsequently dilutional hyponatremia in these patients [4-5]. In beer potomania patients, the ability to reabsorb free-water from the collecting tubules is decreased due to suppressed antidiuretic hormone [2]. This suppression of antidiuretic hormone explains a brisk diuresis in our patients following the administration of solute (0.9% sodium chloride, thiamine, magnesium sulfate, folic acid, multivitamins, chlordiazepoxide) in the emergency rooms as well as a sudden increase in the serum sodium level of these patients over the short period of time.

In the literature review performed by Sanghvi, et al. 18% of the patients with beer potomania developed osmotic demyelination syndrome [4]. Oligodendrocytes are myelinating cells of the central nervous system. They are extremely sensitive to sudden increases in serum sodium level, resulting in pontine or extrapontine demyelination. This process of demyelination is known as osmotic demyelination syndrome (ODS) [2,4,8-9]. ODS can result in dyspnea, dysphagia, dysarthria, and ataxia [10].

Management guidelines

After a detailed review of the literature and understanding the pathophysiology of beer potomania, Sanghvi, et al. [4] suggested the management guidelines to prevent a rapid increase in serum sodium level and development of ODS. Their recommendations are listed below (Table 3).

**Table 3:** Recommendations by Sanghvi, et al. for correction of hyponatremia in beer potomania.

Management Recommendations for Correction of Hyponatremia in Beer Potomania
Nothing by mouth except medications for 24 hours
No intravenous fluids unless symptomatic
Prescribe intravenous fluids in finite amounts if needed
Intensive care status
Check serum sodium every two hours
Goals - Serum sodium increase < 10 mEq/L in first 24 hours - Serum sodium increase < 18 mEq/L in first 48 hours
Reduce serum sodium levels if necessary
Give any intravenous medications in sugar solutions (5% dextrose in water)
If caloric intake is needed, use intravenous sugar solution (5% dextrose in water)

## Conclusions

In this case study, we tried to highlight the importance of the early detection of beer potomania in an alcoholic patient, presenting with severe hyponatremia in the emergency department. This case study was also designed to illustrate the importance of understanding basic pathophysiology of beer potomania, for the successful management and prevention of any neurological sequelae. This case study and our brief review of the literature are directed towards an improvement in the management of beer potomania. In addition, we also find the recommendations suggested by Sanghvi, et al. consistent with the relatively safe management of severe hyponatremia.
